# Multi-phase Training in Virus-Like Particle Synthesis to Foster Science Self-efficacy in Students With Minimal Laboratory Experience

**DOI:** 10.21769/BioProtoc.5381

**Published:** 2025-07-20

**Authors:** Macie A. Proctor-Roser, Marinca Faimau, Julianne Peabody, Krystal R. Charley, Bryce Chackerian, Naomi R. Lee

**Affiliations:** 1 Department of Biology, Northern Arizona University, Flagstaff, AZ, USA; 2 Department of Molecular Genetics & Microbiology, University of New Mexico, Albuquerque, NM, USA; 3 Department of Chemistry & Biochemistry, Northern Arizona University, Flagstaff, AZ, USA

**Keywords:** Peer-to-peer training, Persistence in STEM, Secondary and post-secondary education, Molecular biology

## Abstract

Science self-efficacy describes the confidence individuals have in their ability to accomplish specific scientific practices. Self-efficacy is one factor linked to success and persistence within STEM fields. The purpose of this protocol is to provide research laboratories with effective methods for teaching and mentoring new students in molecular biology, specifically in the synthesis of virus-like particles (VLPs) derived from bacteriophages. VLPs are multivalent nanoparticle structures that can be utilized in multiple biomedical applications, including platforms for vaccine and drug delivery. Production of bacteriophage VLPs using bacterial expression systems is feasible in most laboratory settings. However, synthesizing and characterizing VLPs can be challenging for new researchers, especially those with minimal laboratory experience or a lack of foundational knowledge in molecular biology. To address this, a multi-phase training protocol was implemented to train new students in VLP synthesis, purification, and characterization. This model was optimized for training numerous high school and undergraduate students. By implementing this multi-phase methodology, the students’ confidence in their abilities to perform specific tasks increased and likely enhanced their persistence in STEM.

Key features

• Multi-phase training model for new students.

• Training phases that build to increase science self-efficacy.

• Successful peer-to-peer training.

## Graphical overview


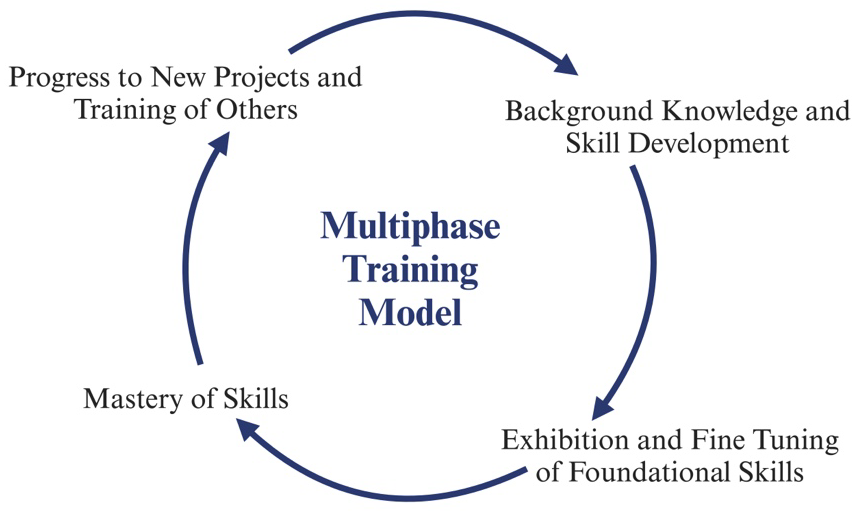


**Multiphase training model: The three-phase process can train students on fundamental molecular biology skills.** Phase I focuses on preparing students for the project by reviewing an individual development plan (IDP) with their mentor, reviewing protocols established by the lab, and reading relevant articles. The first phase concludes with students observing their peers on the protocols and asking questions for clarity. Phase II involves the students conducting hands-on experiments with guidance from a peer. Students may ask for clarity on protocols and mistakes from their peer or supervisor. Students also become familiar with lab-related software. Students transition into phase III when they are confident to work independently. The scale of the experiments also increases with self-efficacy. By the end of Phase II, students can develop an abstract and a poster related to their project.

## Background

Science self-efficacy is an individual’s confidence in their ability to complete specific scientific practices such as research, grant writing, or development of protocols [1]. Self-efficacy is one factor linked to success and persistence within STEM fields among aspiring scientists, especially racial/ethnic minority students [2]. It is important for students with minimal laboratory experience to build their science self-efficacy to increase their likelihood of persistence in STEM. We describe a multi-phase model designed to build students' confidence within each phase, adding to their overall science self-efficacy. This model is implemented among high school and undergraduate students with little or no laboratory experience.

**Figure 1.**
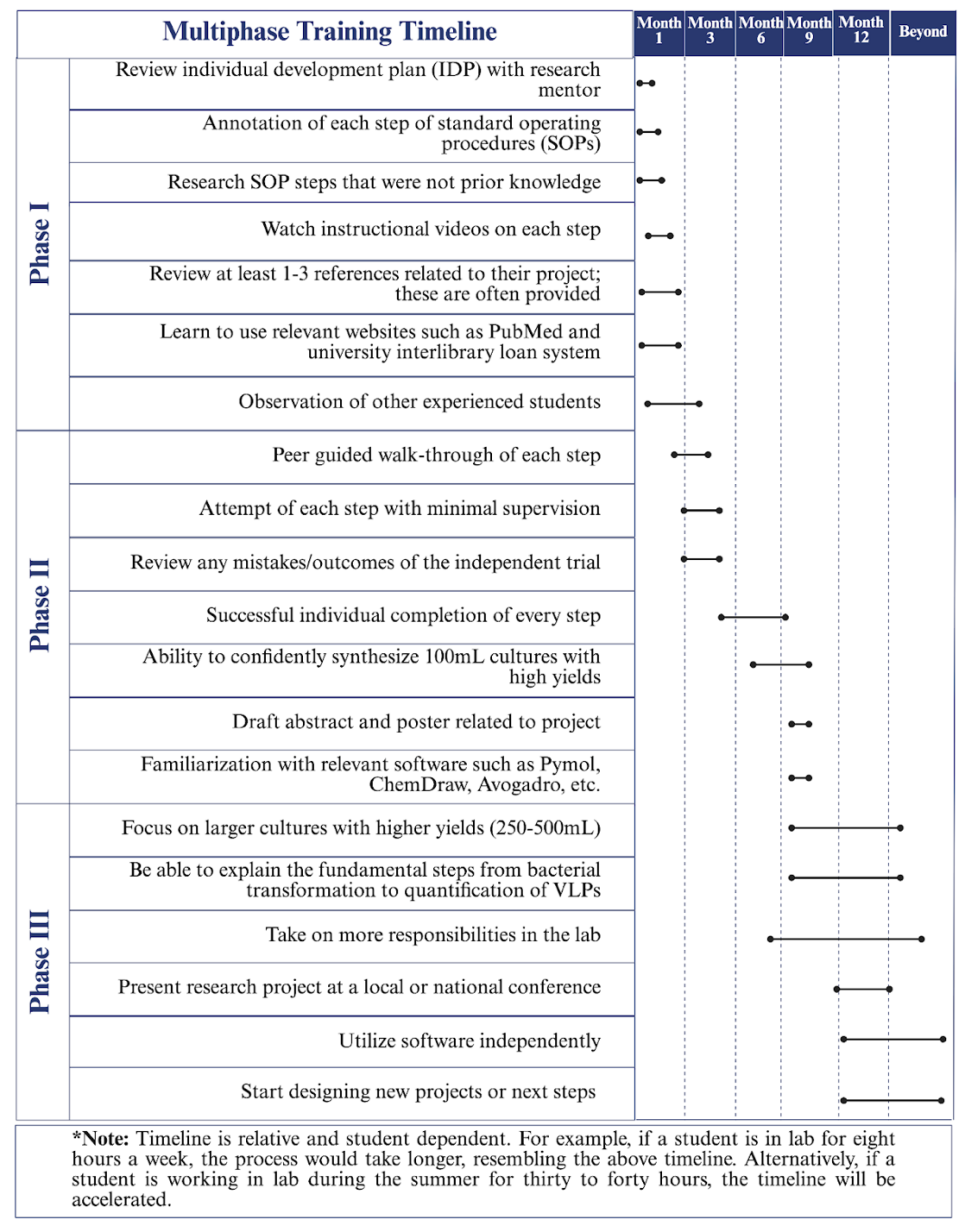
Multiphase training timeline. The three-phase training timeline can take up to one academic year. Phase I focuses on required training such as IDP development, SOP reviews, and instructional guidance review. Phase II relies on peer guidance through the protocol to produce VLPs and to prepare presentations. Finally, Phase III promotes individual completion of the protocol with high production of VLPs and the presentation of their work.

Phase I is focused on building strong foundations through the fundamentals of research (Graphical overview). Once students have a fundamental understanding of the research they are working on, they progress to Phase II. Within Phase II, students work on developing their skills through hands-on learning experiences, allowing them to familiarize themselves with instruments utilized for their research. Therefore, it is not uncommon for students to make mistakes during the learning process. Students can learn from making mistakes and recognize how learning from mistakes is a crucial aspect of developing critical thinking and problem-solving skills. Phase II concludes with students demonstrating their ability to produce and purify proteins by growing small bacterial cultures (100 mL) of virus-like particles (VLPs) with high yields and purifying the VLPs by size-exclusion chromatography. Phase III promotes confidence in students' skills, preparing them to lead peer-to-peer training for incoming students, present at conferences, and develop and fine-tune their skills for future research opportunities. This approach fosters scientific fluency and confidence and can be adapted to any type of bench-based research. Phases I and II require at least a semester or 15 weeks of training. Ultimately, students can successfully complete all three phases within one academic year (Figure 1).

This protocol explains how to implement the multi-phase method using the synthesis and purification of virus-like particles (VLPs) as a model activity. VLPs consist of viral structural proteins that, when overexpressed, spontaneously self-assemble into particles that resemble the virus they are derived from [3]. Importantly, because VLPs do not contain viral genetic material, they are completely non-infectious. VLPs resemble infectious virions and have been used as vaccines for several viruses, including human papillomavirus (HPV) and hepatitis B virus (HBV). Their highly repetitive and multivalent structures render them highly immunogenic, making them suitable as vaccine platforms for targeting diverse molecules, including antigens from pathogens, self-molecules involved in chronic diseases, and even drugs of abuse [3–5]. VLPs have also been used in other biomedical applications, including drug delivery [5]. While VLPs are produced in a variety of expression systems, including mammalian cells, insect cells, and yeast, production of VLPs derived from bacteriophages using an *E. coli* bacteria-based expression system is accessible to most laboratory settings.

**Figure 2.**
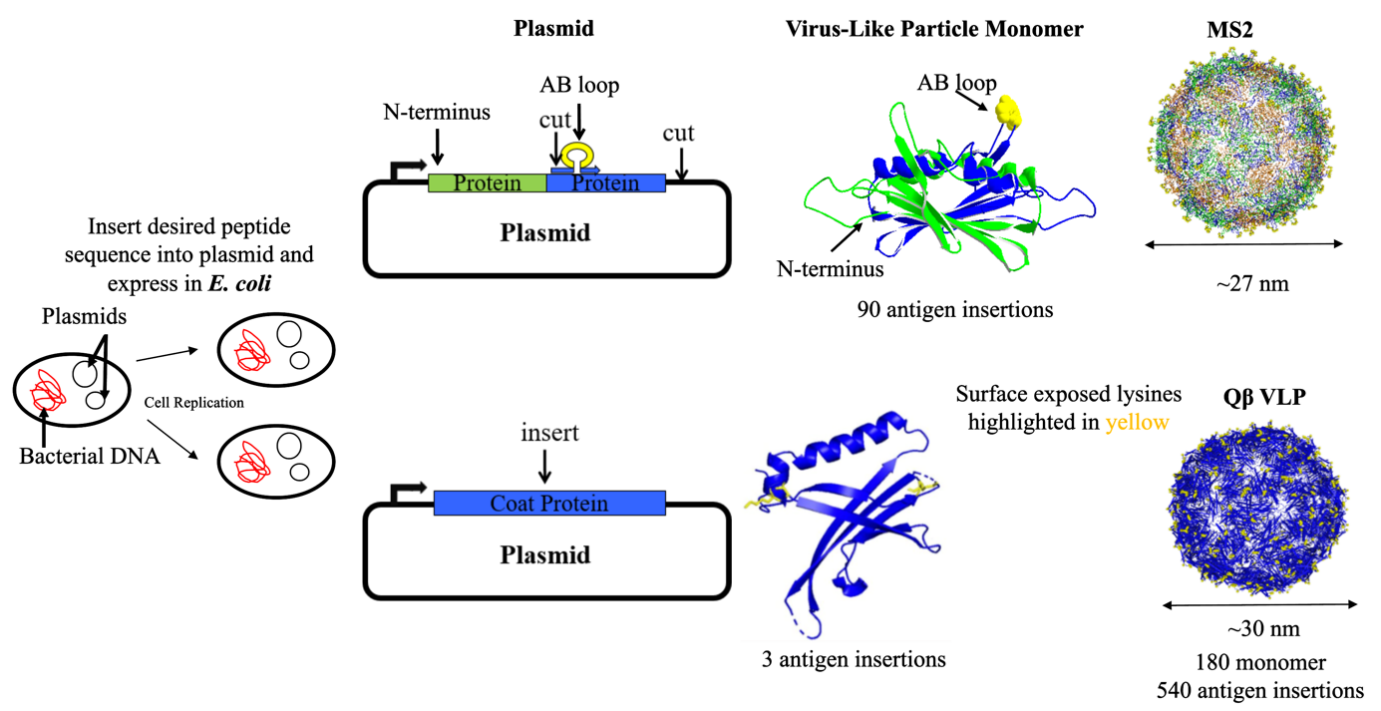
Synthesis pathway of Qβ and MS2 virus-like particles (VLPs). This figure illustrates the engineering of VLP plasmids and their expression in *E. coli* through bacterial transformation. The MS2 VLP plasmid (top) includes a human papillomavirus (HPV) antigen inserted at the N-terminus of the single-chain dimer of the coat protein. These coat proteins self-assemble into ~27 nm VLPs displaying the HPV antigen, with potentially 90 antigen insertion sites. The Qβ VLP plasmid (bottom) features the insertion of a lysine amino acid into the coat protein. These coat proteins self-assemble into ~30 nm VLPs that display lysines (highlighted in yellow) on their surface with potentially 540 antigen insertion sites.

VLPs derived from a family of single-stranded RNA (ssRNA) bacteriophages, including AP205, Qβ, and MS2, have been widely used as vaccine platforms due to their ability to display diverse vaccine targets [6,7]. MS2 VLPs consist of a single structural coat protein that dimerizes and self-assembles into a ~27 nm diameter particle composed of 90 coat protein dimers (Figure 2A). In contrast, Qβ VLPs assemble into pentamers and hexamers, stabilized through disulfide linkages to form a ~30 nm diameter particle made up of 180 coat protein monomers, each with surface-exposed lysines, making up 540 antigen attachment sites (Figure 2B). There are multiple ways to display antigens on the surface of bacteriophage VLPs [6,8,9], with the two most common being genetic engineering and chemical conjugation. However, this manuscript focuses on the synthesis and purification of unmodified VLPs, primarily Qβ, though the protocol can be utilized for various research areas.

## Materials and reagents

**Biological materials**

1. OverExpress^TM ^C41(DE3) chemically competent cells (Sigma-Aldrich, catalog number: CMC0017)

2. *E. coli* 10G ELITE^®^ SixPack Electrocompetent Cells (Biosearch Technology, catalog number: 60052-3)

3. Q-beta and pDSP62 plasmids (obtained from the Chackerian Lab at the University of New Mexico)

**Reagents**

1. 2-Mercaptoethanol (Sigma-Aldrich, catalog number: M3148)

2. 2-(N-morpholino)ethanesulfonic acid (MES) free acid (Gold Bio, catalog number: M-095-1)

3. Ammonium sulfate (Sigma-Aldrich, catalog number: A4418)

4. Bacto yeast extract (Thermo Fisher Scientific, catalog number: 212750)

5. Bromphenol blue (Sigma-Aldrich, CAS number: 115-39-9)

6. Certified molecular biology agarose (Bio-Rad, catalog number: 1613102)

7. Coomassie blue R-250 (Thermo Fisher Scientific, CAS number: 6104-59-2)

8. DNase 1 (Thermo Fisher Scientific, catalog number: 11284932001)

9. Ethylenediaminetetraacetic acid disodium salt dihydrate (EDTA) (Sigma-Aldrich, catalog number: E9884)

10. UltraPure^TM^ ethidium bromide, 10 mg/mL (Thermo Fisher Scientific, catalog number: 15585011)

11. Acetic acid, glacial (Sigma-Millipore, catalog number: AX0073-75)

12. Glycerol (Thermo Fisher Scientific, CAS number: 56-81-5)

13. ImMedia^TM^ Kan agar (Thermo Fisher Scientific, catalog number: 45-0043)

14. Isopropyl β-D-thiogalactopyranoside (IPTG) (Thermo Fisher Scientific, catalog number: 15529019)

15. Kanamycin disulfate salt from *Streptomyces kanamyceticus* (Sigma-Aldrich, catalog number: K1876)

16. Lysozyme from chicken egg white (HEL) (Sigma-Aldrich, catalog number: L6876)

17. Magnesium chloride solution (Sigma-Aldrich, catalog number: 68475)

18. Magnesium sulfate solution (Sigma-Aldrich, catalog number: M3409)

19. Orange G (Sigma-Aldrich, catalog number: O3756)

20. Tris base (Sigma-Aldrich, catalog number: 648310-M)

21. Potassium chloride (Fisherbrand, catalog number: P217-500)

22. Potassium phosphate monobasic (Fisherbrand, catalog number: P285-500)

23. SeeBlue^TM^ Pre-stained standard (1×) (Invitrogen, catalog number: LC5625)

24. Sodium acetate (Sigma-Aldrich, catalog number: S2889)

25. Sodium chloride (Fisherbrand, CAS number: 7647-14-5)

26. Sodium dodecyl sulfate (SDS) (Sigma-Aldrich, catalog number: L3771)

27. Sodium phosphate dibasic (Fisherbrand, CAS number: 7558-79-4)

28. Tris-base (Sigma-Aldrich, catalog number: 648310-M)

29. Tris (1 M, pH 8.4) (Alfa Aesar, CAS number: 77-86-1)

30. Tris-HCl (1 M, pH 7.5) (Fisherbrand, catalog number: BP1757)

31. Tryptone (Sigma-Millipore, catalog number: T9410)

32. Sodium hydroxide (NaOH) pellet 500 G AR^®^ (ACS) (Thomas Scientific, catalog number: C818M3)

**Solutions**

*Note: The “×” unit (i.e., 1×) indicates the concentration of a solution.*

1. 5× loading dye (for loading protein onto an acrylamide gel) (see Recipes)

2. 6× orange loading dye (for loading DNA onto an agarose gel) (see Recipes)

3. 1% agarose gel (see Recipes)

4. 10% deoxycholate solution (DOC) (see Recipes)

5. 70% ethanol solution (see Recipes)

6. 1× 2-(N-Morpholino)-ethanesulfonic acid buffer (MES) (see Recipes)

7. 1× phosphate-buffered saline (PBS) (see Recipes)

8. 1× SCB (see Recipes)

9. 1× tris-acetate EDTA (TAE) (see Recipes)

10. 0.5 M ethylenediaminetetraacetic acid disodium salt dihydrate solution (EDTA) (see Recipes)

11. 5 M sodium chloride solution (see Recipes)

12. Coomassie blue low-toxicity staining solution (for staining acrylamide gel) (see Recipes)

13. 10 mg/mL DNase solution (see Recipes)

14. 100 mg/mL kanamycin solution (see Recipes)

15. Isopropyl β-D-thiogalactopyranoside solution (IPTG) (see Recipes)

16. Lysogeny broth (LB) (see Recipes)

**Recipes**

diH_2_O = deionized water

dH_2_O = distilled water

UPW = ultrapure water

**1. 5× loading dye**

*Note: For loading protein onto an SDS PAGE gel electrophoresis. It contains SDS, which is an irritant. Avoid inhalation and skin contact.*

**Table:** 

Reagent	Final concentration	Quantity or volume
Tris-HCl (1 M, pH 6.8)	0.2 g/L	2 mL
Sodium dodecyl sulfate	277 mM	0.8 g
Glycerol	4.90 M	4 mL
β-mercaptoethanol	570 mM	0.4 mL
Bromophenol blue	1.2 mM	8 mg
diH_2_O	n/a	3.6 mL
Total	n/a	10 mL

a. Measure all the reagents and diH_2_O into a 100 mL graduated glass cylinder.

b. Mix vigorously using a vortex mixer to dissolve reagents.

c. Aliquot into 1.5 mL microcentrifuge tubes.

d. Store at -20 °C. Stable for one year under these conditions.

**2. 6× Orange DNA loading dye**

*Note: For loading DNA onto an agarose gel electrophoresis.*

**Table:** 

Reagent	Final concentration	Quantity or volume
TAE (50×)	3.10 M	2 mL
Orange G	0.033 M	0.15 g
Glycerol	0.00407 M	60 mL
dH_2_O	n/a	38 mL
Total	3.13 M	100 mL

a. Measure all the reagents and diH_2_O into a 100 mL graduated glass cylinder.

b. Mix vigorously using a vortex mixer to dissolve reagents.

c. Aliquot into 1.5 mL microcentrifuge tubes.

d. Store at room temperature. Stable for one year under these conditions.

**3. 1% agarose gel (100 mL)**

*Note: When handling ethidium bromide, open the bottle facing AWAY from you to avoid eye/skin contact. When heating/cooling the solution, avoid inhaling the steam. Ethidium bromide is highly toxic and an irritant.*

**Table:** 

Reagent	Final concentration	Quantity or volume
TAE (1×)	n/a	100 mL
Agarose powder	n/a	1 g
Ethidium bromide	0.0001 mg/mL	1 µL
Total	1 g/100 mL	100 mL

a. Measure all the reagents, except ethidium bromide, into a 200 mL glass Erlenmeyer flask.

b. Microwave for 1 min 30 s.

c. Cool solution in a fume hood until warm to the touch.

d. Add ethidium bromide.

e. Pour into a gel mold to let it cool in a 4 °C fridge until it settles.

**4. 10% deoxycholate solution (DOC) (100 mL)**

*Note: Solution decomposes when exposed to ‘direct light. Perform in a room with minimal lighting.*

**Table:** 

Reagent	Final concentration	Quantity or volume
Sodium deoxycholate	10 g/100 mL	10 g
dH_2_O	n/a	100 mL
Total	10 g/100 mL	100 mL

a. Wrap the bottle with aluminum foil, neck to bottom, to avoid any light contact.

b. Place a magnetic stir bar in the bottle.

c. Add 80 mL of dH_2_O and 10 g of sodium deoxycholate.

d. Immediately close the lid and stir the solution on a magnetic stir plate.

e. Let it stir for 10–15 min.

f. Store at room temperature. Stable for one year under these conditions.

**5. 70% ethanol solution (100 mL)**

*Note: Pure ethanol is highly flammable and an irritant. Avoid handling near flames and direct contact with the skin.*

**Table:** 

Reagent	Final concentration	Quantity or volume
Ethanol (100%)	70%	70
dH_2_O	n/a	30 mL
Total	70 g/100 mL	100 mL

a. Measure all the reagents and dH_2_O into an amber/brown bottle.

b. Invert the bottle a few times to mix the solution.

c. Store at room temperature.

**6. Coomassie Blue low-toxicity staining solution**

*Note: Solution may stain skin and clothing. Wear gloves and a lab coat for precaution.*

**Table:** 

Reagent	Final concentration	Quantity or volume
Ethanol (95%)	0.027 M	500 mL
Coomassie Blue R-250 [0.1% (w/w)]	0.0012 M	1.25 g
Glacial acetic acid [99.7% (w/w)]	0.111 M	125 mL
dH_2_O	n/a	500 mL
Total	0.139 M	1,250 mL

a. Measure Coomassie blue and ethanol in a 2 L graduated glass cylinder.

b. Add 500 mL of diH_2_O.

c. Dissolve the dye completely by sealing the opening and inverting the glassware to mix.

d. Add glacial acetic acid, seal the opening, and invert the glassware to mix.

e. Bring volume up to a total of 1,250 mL with dH_2_O and invert to mix for 5 min.

f. Store at room temperature. Stable for one year under this condition.

**7. 2-(N-Morpholino) ethanesulfonic acid buffer (1 L)**

*Note: MES buffer is used for running acrylamide gels (for protein). It contains SDS, which is an irritant. Avoid inhalation and skin contact.*

**Table:** 

Reagent	Final concentration	Quantity or volume
2-(N-Morpholino) ethanesulfonic acid	0.04 M	9.76 g
Tris base	1.0 M	6.06 g
Sodium dodecyl sulfate	0.0693 M	1.0 g
EDTA	0.0205 M	0.3 g
diH_2_O	n/a	1 L
Total	1.1298 M	1 L

a. Measure all the reagents and diH_2_O into a 1 L Duran bottle.

b. Add 500 mL of diH_2_O.

c. Dissolve completely by sealing the opening, adding a magnetic stir bar, and letting it stir on a magnetic stirrer.

d. Bring volume up to a final volume of 1 L with diH_2_O and mix. Remove the stir bar.

e. Store at room temperature. Stable for one year under this condition.

**8. Phosphate-buffered saline (PBS) (20 L)**

*Note: Allow the reagents to dissolve completely in 500 mL of UPW before adding any more UPW. Store at room temperature.*

**Table:** 

Reagent	Final concentration	Quantity or volume
Sodium chloride	136 mM	160 g
Potassium chloride	2 mM	4 g
Sodium phosphate dibasic	8.1 mM	23 g
Potassium phosphate monobasic	1.46 mM	4 g
Ultrapure water (UPW)	n/a	20 L
Total	147.56 mM	20 L

a. Fill a 20 L jug with UPW.

b. Remove 1 L from the jug into a separate glassware.

c. Measure all the reagents above and add to the 1 L UPW.

d. Dissolve completely by sealing the opening, adding a magnetic stir bar, and letting it stir on a magnetic stirrer.

e. Remove the stir bar and pour the dissolved solution into the 20 L jug.

f. Mix the solution in the jug by rocking it back and forth.

g. Store at room temperature. Stable for one year under this condition.

**9. SCB buffer (1 L)**

*Note: Allow the reagents to dissolve completely in 500 mL of diH_2_O before adding any more diH_2_O. Store at 4 °C.*

**Table:** 

Reagent	Final concentration	Quantity or volume
Tris-HCl (1 M, pH 7.4)	0.01 M	10 mL
Sodium chloride solution (5 M)	0.1 M	20 mL
Magnesium sulfate solution (1 M)	0.001 M	1 mL
diH_2_O	n/a	969 mL
Total	0.111 M	1 L

a. Measure all the reagents into a 1 L Duran bottle.

b. Add 500 mL of diH_2_O.

c. Dissolve completely by sealing the opening, adding a magnetic stir bar, and letting it stir on a magnetic stirrer.

d. Bring volume up to a final volume of 1 L with diH_2_O and mix. Remove the stir bar.

e. Store at 4 °C. Stable for one year under this condition.

**10. TAE buffer (1 L of 1×)**

*Note: TAE (Tris-Acetate-EDTA) is the most common buffer used for agarose gel electrophoresis for the analysis of nucleotides or DNA.*

**Table:** 

Reagent	Final concentration	Quantity or volume
Tris-base	40 mM	4.844 g
Sodium acetate	5 mM	0.41 g
EDTA	2 mM	0.7445 g
Glacial acetic acid [99.7% (w/w)]	33.6 mM	1.93 mL
diH_2_O	n/a	1 L
Total	80.6 mM	1 L

a. Measure all the reagents and diH_2_O into a 1 L Duran bottle.

b. Add 500 mL of diH_2_O.

c. Dissolve completely by sealing the opening, adding a magnetic stir bar, and letting it stir on a magnetic stirrer.

d. Bring volume up to a final volume of 1 L with diH_2_O and mix. Remove the stir bar.

e. Store at 4 °C. Stable for one year under this condition.

**11. 0.5 M EDTA solution (100 mL)**

*Note: Allow the reagents to dissolve completely in 80 mL of dH_2_O and measure the pH level before adding any more dH_2_O. EDTA is an irritant. Avoid skin/eye contact. Wear gloves when handling.*

**Table:** 

Reagent	Final concentration	Quantity or volume
EDTA	0.5 M	18.61 g
Sodium hydroxide (NaOH) pellets	0.5 M	2 g
dH_2_O	n/a	100 mL
Total	1.0 M	100 mL

a. Measure EDTA into 500 mL Duran bottle.

b. Add 80 mL of dH_2_O and adjust the pH to 8.0 with NaOH pellets.

*Note: EDTA will not dissolve completely into solution until pH 8.0.*

c. Once fully dissolved, bring volume up to 100 mL with dH_2_O, add a magnetic stir bar, and let it stir for 5 min.

d. Store in an acid cabinet at room temperature. Stable for one year under these conditions.

**12. 5 M sodium chloride solution (100 mL)**

*Note: Allow the reagents to dissolve completely in 800 mL of dH_2_O before adding any more dH_2_O.*

**Table:** 

Reagent	Final concentration	Quantity or volume
Sodium chloride	5 M	292 g
dH_2_O	n/a	1 L
Total	5 M	1 L

a. Measure sodium chloride and 800 mL of dH_2_O in a 1 L Duran bottle.

b. Dissolve completely by sealing the opening, adding a magnetic stir bar, and letting it stir on a magnetic stirrer.

c. Once fully dissolved, bring volume up to 1 L with dH_2_O and let it stir for 5 min. Remove the stir bar.

d. Store at room temperature. Stable for one year under this condition.

**13. DNase solution (10 mL of 10 mg/mL)**

**Table:** 

Reagent	Final concentration	Quantity or volume
DNase 1	10 mg/mL	100 mg
Nuclease-free H_2_O	n/a	10 mL
Total	10 mg/mL	10 mL

a. Measure DNase 1 and nuclease-free H_2_O in 100 mL glassware.

b. Stir to mix until dissolved.

c. Aliquot in 1.5 mL microcentrifuge tubes.

d. Store at -20 °C. Stable for one year under this condition.

**14. IPTG solution (20 mL)**

**Table:** 

Reagent	Final concentration	Quantity or Volume
IPTG	23.8 mg/mL	0.476 g
diH_2_O	n/a	20 mL
Total	23.8 mg/mL	20 mL

a. Measure IPTG and diH_2_O in 100 mL glassware.

b. Stir to mix until dissolved.

c. Sterilize the solution by filtering through a microcentrifuge spin column.

d. Centrifuge for 1 min at 9,021.68 rcf.

e. Collect filtrate and aliquot in 1.5 mL microcentrifuge tubes.

f. Store at -20 °C. Stable for one year under this condition.

**15. Kanamycin solution (100 mL)**

*Note: Kanamycin is a health hazard. Avoid direct skin contact and inhalation. Wear gloves when handling.*

**Table:** 

Reagent	Final concentration	Quantity or volume
Kanamycin	100 mg/mL	1 g
dH_2_O	n/a	10 mL
Total	100 mg/mL	10 mL

a. Measure kanamycin and dH_2_O in 100 mL glassware.

b. Stir to mix until dissolved.

c. Centrifuge for 1 min at 9,021.68 rcf.

d. Collect filtrate and aliquot in 1.5 mL microcentrifuge tubes.

e. Store at -20 °C. Stable for one year under this condition.

**Media**

Most of the following media should have a final pH of 7.0 ± 0.2, as *E. coli* grows best in a neutral solution. Generally, the role of a base in a growth medium is to bring the solution to a neutral or nearly neutral pH. When no other base is mentioned, use NaOH as necessary.

When doubling ingredients for a medium, but not the final volume, to make a concentrated, rich growth medium (such as 2× LB, a doubly concentrated form of LB), unless otherwise instructed, one should not double the acids or bases, except when necessary for adjusting pH level. Also, one should generally not double the salt.

Media should be aliquoted and autoclaved shortly after being made. If it cannot be autoclaved within a reasonable time (before the end of the day), it should be refrigerated to prevent the growth of contaminants.

**1. LB broth (1 L)**

*Note: LB (an acronym for lysogeny broth, although it is often misinterpreted as Luria Broth, Lennox Broth, or Luria-Bertani medium) was originally developed to optimize* Shigella *growth and plaque formation. It is the standard growth medium for* Escherichia coli *(a relative of* Shigella).

**Table:** 

Reagent	Final concentration	Quantity or volume
Tryptone	140 mM	10 g
Yeast extract	n/a	5 g
Sodium chloride	85.5 mM	5 g
diH_2_O	n/a	1 L
Total	225.5 mM	1 L

a. Measure all the reagents into a 1 L glassware.

b. Add 500 mL of diH_2_O.

c. Dissolve completely by sealing the opening, adding a magnetic stir bar, and letting it stir on a magnetic stirrer.

d. Once fully dissolved, bring volume up to 1 L with dH_2_O and let it stir for 5 min. Remove the stir bar.

e. To make 100 mL, 250 mL, and 500 mL cultures, pour LB solution into an Erlenmeyer Flask as follows:

100 mL of LB solution in a 250 mL flask

250 mL of LB solution in a 500 mL flask

500 mL of LB solution in a 1,000 mL flask

f. Seal the flask opening with aluminum foil and paste a small strip of autoclave tape on the neck of the flask.

g. Autoclave at 121 °C for 1 h.

h. Once cooled, swirl the flask to ensure it is fully mixed.

i. Store at room temperature. Stable for 2–3 months under this condition.

**Laboratory supplies**

1. Bemis^TM^ Parafilm^TM^ M laboratory wrapping film, 4 in. × 125 ft. (Fisher Scientific, catalog number: 13-374-10)

2. Celltreat round bottom centrifuge tube, 50 mL (Fisher Scientific, catalog number: 50-828-732)

3. CryoKING premium cardbox freezer boxes, 3 in., 81 wells (BiologixUSA, catalog number: 90-2381)

4. Culture tubes with closer, 17 × 100 mm, vol. 14 mL (VWR, catalog number: 60818-664)

5. Dialysis tub (VPET Plastics, catalog number: J110CT-128-02)

6. Easy Reader^TM^ conical polypropylene centrifuge tubes, 15 mL (Fisher Scientific, catalog number: 05-539-4)

7. Easy Reader^TM^ conical polypropylene centrifuge tubes, 50 mL (Fisher Scientific, catalog number: 339653)

8. GeneMate graduated microcentrifuge tubes, 1.7 mL (VWR, catalog number: 490004-436)

9. Invitrogen^TM^ gel knife (Thermo Fisher Scientific, catalog number: EI9010)

10. Gene Pulsers/MicroPulser^TM^ cuvettes, 0.1 cm gap (Bio-Rad, catalog number: 165-2089)

11. Nichrome/aluminum inoculating loops (Fisher Scientific, catalog number: 13-104-5)

12. Nonpyrogenic serological pipette, 5 mL (Fisher Scientific, catalog number: 13-678-11D)

13. Nonpyrogenic serological pipette, 10 mL (Fisher Scientific, catalog number: 13-678-11E)

14. Nonpyrogenic serological pipette, 25 mL (Fisher Scientific, catalog number: 13-678-11)

15. NuPAGE^TM^ 10%, Bis-Tris, 1.0–1.5 mm, mini protein gels (Thermo Fisher Scientific, catalog number: NP0302BOX)

16. Pierce^TM^ protein concentrator PES, 100 MWCO, ≤ 0.5 mL (Thermo Fisher Scientific, catalog number: 88503)

17. Pierce^TM^ protein concentrator PES, 100 MWCO, 2–6 mL (Thermo Fisher Scientific, catalog number: 88523)

18. Pierce^TM^ protein concentrator PES, 100 MWCO, 5–20 mL (Thermo Fisher Scientific, catalog number: 88533)

19. Petri dish, slippable lid 100 mm × 15 mm, sterile, polystyrene (Fisherbrand, catalog number: FB0875713)

20. Plastic cuvettes, 1–1.5 mL (UV-VIS) (Vernier, catalog number: cuv-uv)

21. Snakeskin^TM^ dialysis tubing (Thermo Fisher Scientific, catalog number: 68035)

22. Snakeskin^TM^ dialysis tubing clips (Thermo Fisher Scientific, catalog number: 68011)

23. TipOne pipette tips, 10 μL (USA Scientific, catalog number: 1111-3000)

24. TipOne pipette tips, 300 μL (USA Scientific, catalog number: 1110-9000)

25. TipOne pipette tips, 1,250 μL (USA Scientific, catalog number: 1112-1020)

26. Octagonal stirring bar kit PTFE (Dynalon Labware, catalog number: 303595)

## Equipment

1. Barnstead^TM^ Genpure^TM^ Pro water purification system (Thermo Fisher Scientific, catalog number: 50131948)

2. Biowave cell density meter CO8000 (Avantor, model: WPA CO8000, catalog number: 80-3000-45)

3. Branson Ultrasonic^TM^ Sonifier^TM ^SFX250 cell disruptors (Fisher Scientific, catalog number: 15-345-138)

4. Compact digital rocker, 100–240 V, 50/60 Hz, US plug (Fisher Scientific, catalog number: 88880019)

5. Digital dry bath (Bio-Rad, catalog number: 1660562EDU)

6. FisherBiotech transilluminator (Fisher Scientific, model: DLT)

7. Xcell Surelock^TM^ mini-cell (Invitrogen, catalog number: EI0001)

8. Magnetic stirrer (ANZESER, catalog number: B07J59QVGQ)

9. MaxQ 4000 incubated shaker (Thermo Fisher Scientific, model: SHKE4000-7)

10. MicroPulser^TM^ (Bio-Rad, model: 411BR 12141)

11. Mini-Sub^® ^cell GT cell (Bio-Rad, model: Mini-Sub^®^ Cell GT)

12. Model 2110 fraction collector (Bio-Rad, catalog number: 7318122)

13. mySPIN^TM^ 12 mini centrifuge (Thermo Fisher Scientific, catalog number: 75004081)

14. MYTEMP^TM^ mini digital incubator (Benchmark Scientific, model: H2200-H)

15. Ohaus Adventurer^TM^ analytical balance (Ohaus, catalog number: AR1140)

16. PowerPac^TM^ basic power supply (Bio-Rad, catalog number: 1645050)

17. Revco^TM^ UxF -86 °C upright ultra-low temperature freezers (Fisher Scientific, model: UXF60086D)

18. Sorvall legend XTR centrifuge (Thermo Fisher Scientific, catalog number: 75004521)

19. Sorvall LYNX 4000 superspeed centrifuge (Thermo Fisher Scientific, catalog number: 75006580)

20. Variable speed vortex mixer (Fisher Scientific, catalog number: 14-955-163)

21. Wide Mini-Sub^®^ cell GT cell (Bio-Rad, model: Wide Mini-Sub^®^ Cell GT)

## Software and datasets

1. EnCor Biotechnology Inc., ammonium sulfate calculator: https://www.encorbio.com/protocols/AM-SO4.htm (access date, 9/10/2023)

## Procedure

**Part I. Foundations of practice and professional growth**

**A. Phase I: Background knowledge and development of skills**

At the start of each semester, students can complete an individual development plan (IDP) and discuss project goals with their research mentor (Graphical overview; also, see Supplementary information, Individual Development Plan [File S1]) [10]. After completing an IDP, students review the lab's standard operating procedures (SOPs) for synthesizing virus-like particles (VLPs) (Figure 2; also see Supplementary information, Agarose Gel Electrophoresis [File S2], Individual Development Plan [File S1], Dialysis [File S3], Growing, Inducing, and Lysis [File S4], SDS-PAGE Electrophoresis [File S5], Size Exclusion Chromatography [File S6], and Transformation [File S7]).

1. Review standard operating procedures (SOPs). SOPs are provided by the laboratory. SOPs for the synthesis of virus-like particles (VLPs) can be found in the Supplemental information section.

a. Students must read through each step carefully before entering the lab setting. This ensures that students have a foundational knowledge of the protocol they will be learning.

b. It is important to give the student access to their lab notebook or some sort of notebook so that they have one space to organize all questions, ideas, and notes.

2. Students should annotate each step of the process from transformation to purification.

a. After students have read through the SOPs and have reviewed each step, they will then annotate and create a note system for the protocol.

b. Students will identify the reagents and the location for all materials, provided that all required lab trainings are completed.

3. Students should research anything that was not previous knowledge.

a. Students will research any jargon, techniques, and concepts prior to entering the laboratory setting.

4. Students should watch instructional videos on each step.

a. These can be provided by the PI or can be found through online resources such as JoVE, YouTube, etc.

5. Students are encouraged to research more about VLPs to strengthen their background knowledge.

6. Students should observe other experienced students.

a. A more advanced peer will begin by talking through each step and explaining in detail what occurs during each procedure and why. The peer will answer any preexisting questions about the procedures.

b. At this stage, the peer will also give a guided walkthrough and tour of the lab, allowing the student to gain knowledge of the location of all safety and laboratory equipment.

c. The student will then observe a peer’s trial to learn technical skills, ask questions, take detailed notes, and establish a basic understanding of the SOPs.

**Figure 3.**
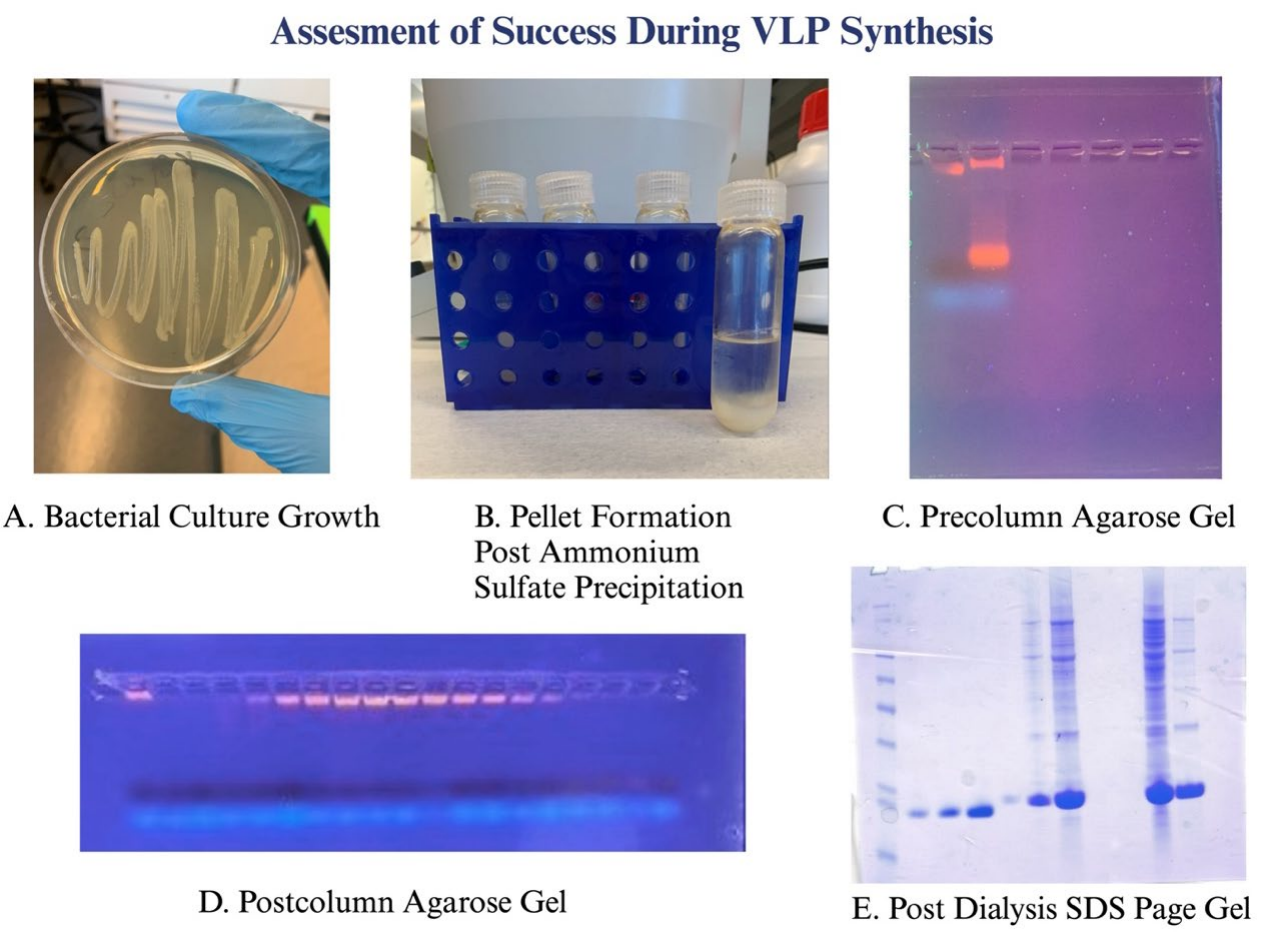
Various checkpoints to assess the success of the experiment. (A) Overnight bacterial growth LB streak plates confirm a successful transformation step. (B) Pellet formation after ammonium sulfate precipitation confirms the breakdown of impurities in the sample. (C) 1% agarose gel run prior to size exclusion chromatography to confirm the sample is intact and ready for running on the column. (D) Post-column 1% agarose gel is run to identify the location of VLPs from the column and that they have not fallen apart. (E) After the VLPs are dialyzed, an SDS-PAGE gel is run to identify concentration levels of purified VLPs.

**B. Phase II: Exhibition and fine-tuning of foundational skills**

Phase II commences with a peer-guided walk-through from beginning to end. Students will complete each step alongside their peer trainer. Upon finishing Phase II, students will be able to confidently synthesize and purify VLPs from small bacterial cultures (100 mL) with high yields.

1. Peer-guided walk-throughs of each step:

a. Students will work through a hands-on guided walkthrough alongside their peer. This assists in developing the proper hand technique for each step involved in the SOPs.

2. Attempt each step with minimal supervision:

a. Students will complete a full trial from start to finish with minimal to no help from their peer. This step teaches students how to outsource, problem solve, and become independent within the lab.

b. Here, students should try not to ask questions and problem-solve on their own unless it is completely necessary.

3. Review any mistakes/outcomes of following the procedure independently.

a. Students and their peers will work through their independent trials to review their accomplishments and things they need to work on. This step is crucial regardless of trial success.

b. This step establishes what went wrong, how to learn from mistakes, and places to identify success or failure. By working through these ideas with a positive mindset and attitude, the students will have the opportunity to build their confidence.

4. Complete procedure with peers to confirm abilities.

a. After individual trials, the students must do at least one more trial with their peer to confirm their ability to move on and work independently.

5. Successful individual completion of every step:

a. Students will be able to demonstrate their abilities to correctly perform their experiment and increase more purified and concentrated yields.

6. Students will discuss with their PI if they should move on or continue to fine-tune their skills with 100 mL cultures.

**C. Phase III: Mastery of skills, preparation for future projects, and training of others**

During Phase III, students will be able to synthesize larger cultures with a high-yield outcome. For our laboratories, we transition to 250 mL and then 500 mL bacteria cultures. The students will continue to solve problems alongside peers but will regularly meet with their research mentor. Here, students will fully understand the fundamental steps from bacterial transformation to quantification of VLPs (Figure 3). Students gain the ability to progress and prepare to train future students. This prepares the students to move forward onto new projects. Students should also be using relevant software independently. Students should also present their current knowledge via oral or poster presentations at local and national meetings. Many students begin by presenting their research at a spring undergraduate symposium by the end of their first or second semester in the lab. Phase III is completed when the students can demonstrate the abilities stated above in a fully conducive manner.

1. Attempt to synthesize cultures with higher yields (250–500 mL).

a. Students will continue to build and develop their skills by moving on to higher yields.

b. Students will begin with 100 mL and then progress to 250 mL and 500 mL cultures.

2. Update and meet with PI to discuss progress.

a. Before moving on to a higher yield, the students must meet with their PI to discuss development, strengths, and places to improve.

b. The PI must decide whether to move the students forward or remain at the same place.

3. Successfully demonstrate all laboratory abilities.

4. Prepare to move on to new projects and continue to fine-tune skills in preparation to become a peer mentor.

**Part II. Synthesis of virus-like particles**

**A. Transformation of electrocompetent C41 *E. coli* cells**

*Note: Prior to starting, ensure you have ice, cuvettes, cells, and autoclaved LB broth. Kan-LB agar plates can be made during the recovery step if not available in advance. Keep cells on ice at all times until electroporation is complete.*

1. Thaw electrocompetent OverExpress^TM^ C41 cells on ice.

a. Prechill a MicroPulser^TM^ cuvette on ice.

2. Transfer 25 µL of thawed C41 cells directly into the reservoir of the prechilled cuvette.

3. Add 1 µL (32.3 fmol) of the plasmid of interest (i.e., Q-beta or MS2) into the reservoir of the prechilled cuvette.

4. Flick the cuvette gently to mix.

5. Set MicroPulser^TM ^to *Bacteria* on *EC1*.

6. Wipe off residual water from the cuvette before electroporation.

7. Immediately add 1 mL of LB broth and pipette up and down twice gently.

8. Transfer culture into a culture tube.

9. Incubate for 1 h at 37 °C and 200 rpm in a shaking incubator.

10. Pipette 25 µL of the culture onto a Kanamycin-treated LB agar plate.

11. Incubate the plate overnight at 37 °C for 12–16 h.

12. Check the plate in the morning for colonies (Figure 3).

13. Seal with Parafilm^TM^ and store at 4 °C.

a. Restreak plates every 2 weeks to keep cultures viable.

**B. Growing, inducing, and lysing cells for VLP production**

*Note: Prior to starting, ensure you have autoclaved LB broth available with the appropriate antibiotic. This process should start in the late afternoon or early evening.*

1. Growth and induction

a. Add 50 µL of Kanamycin (100 mg/mL) to a 100 mL LB broth Erlenmeyer flask.

b. Transfer 5 mL of the LB broth into a culture tube.

*Note: Save the remaining LB broth for step B1e.*

c. Add 1 colony of transformed C41 *E. coli* cells (Part II, Section A).

d. Incubate overnight at 37 °C and 200 rpm in a shaking incubator for 12–16 h.

e. Add overnight culture into the same 100 mL LB broth flask from step B1a.

f. Place culture flask back into the shaking incubator at 37 °C and 200 rpm. Monitor optical density (OD) every hour until it reaches 0.6–1.0.

*Note: OD doubles every 30 min.*

g. Add 400 µL of IPTG.

h. Incubate in a shaking incubator for 3 h at 37 °C and 200 rpm in a shaking incubator.

i. Decant culture into a centrifuge bucket.

j. Centrifugate for 20 min at 3,286 rcf at 4 °C.

k. Discard supernatant and freeze pellets at -80 °C overnight (12–16 h).

*Note: Pellets are viable indefinitely under this condition.*

2. Lysis

a. Thaw pellets stored at -80 °C on ice.

b. Add 10 mL of lysozyme buffer (without DTT) and vortex until pellets dissolve.

c. Add 50 µL of 10% deoxycholate.

d. Incubate on ice for 30 min.

e. Transfer contents to a 50 mL conical tube for sonication.

*Note: Sonicate at ~25% for 10 s (repeat 5 times).*

f. Add 100 µL of MgCl2 and 20 µL of DNase (10 mg/mL).

g. Incubate on ice for 1 h.

h. Centrifuge for 30 min at 3,286 rcf at 4 °C.

i. Collect supernatant and transfer to a new tube.

j. Add ammonium sulfate using the ammonium sulfate calculator from EnCor Biotechnology Inc., at https://www.encorbio.com/protocols/AM-SO4.htm.

i. Temperature of 4 °C.

ii. Starting volume of solution in mL: volume of supernatant.

iii. Desired % saturation: 70.

iv. Starting % saturation: 0.

k. Store samples at 4 °C overnight (12–16 h).

*Note: Samples can be stored at 4 °C for short-term storage (1 week).*

l. Centrifuge for 10 min at 11,648 rcf.

m. Discard supernatant and resuspend with 5 mL of 1× SCB buffer.

n. Centrifuge for 10 min at 11,648 rcf.

o. Collect supernatant into a 15 mL conical centrifuge tube.

p. Test supernatant on a 1% agarose TAE gel (Figure 3). See Section B6 for instructions.

3. Size Exclusion Chromatography

*Note: Prior to starting, ensure solvent reservoir of the size exclusion chromatography is filled appropriately with 1× SCB buffer. Ensure the pump is submerged in buffer.*

a. Initiate flow of 1× SCB buffer through column.

b. Let the column flush for 30 min.

c. Stop the flush, and pipette out the remaining layer of buffer at the top of the column.

d. Carefully load sample from lysis step to the top of the column without disrupting the Sepharose resin.

e. Initiate flow of 1× SCB buffer through the column.

f. Start the fraction collector and ensure drops are collected into the tube.

g. Return after 30 min to ensure column is still flowing and fraction tubes are collecting droplets.

*Note: Collection should run for ~8 h.*

*Follow-up*

a. After 8 h, stop the flush, and remove the carousel from fraction collector.

*Note: Carousel can be sealed stored in 4 °C while preparing an agarose gel.*

b. Prepare 1% agarose TAE gel (Figure 3). See Section B6 for instructions.

c. Test samples from every other tube starting from tube 10.

*Note: VLPs typically appear between tubes 10 and 36.*

d. Pool fractions that indicate positive for VLPs.

*Note: Collect before the first positive and after the last positive (i.e., if 10–26 are positive, collect from tubes 9 to 27).*

e. Add ammonium sulfate (see Section B2 for instructions).

f. Store at 4 °C overnight (12–16 h).

*Note: Samples can be stored at 4 °C for short-term storage (1 week).*

4. Dialysis for buffer exchange

a. Centrifuge the overnight sample from the previous step (step B3f) for 10 min at 11,648 rcf.

b. Discard supernatant and resuspend pellets with 1 mL of 1× PBS.

c. Trim 4 inches of Snakeskin^TM^ dialysis tube and soak in 1× PBS for 15 s.

d. Clip one end of the dialysis tube with a dialysis clip.

e. Transfer sample into dialysis tube, ensure no air bubbles are present, and clip the opening with another dialysis clip.

f. Place the sample and magnetic stir bar in a tub filled with 2.5 L of 1× PBS.

g. Turn over the tub and place on a stirring plate.

*Note: Adjust stirring rate to ensure consistent spinning without the sample contacting the stir bar.*

h. Run dialysis for 24 h.

i. Replenish with 2.5 mL of 1× PBS and continue dialysis for another 24 h.

j. Collect sample into 1.5 mL microcentrifuge tubes.

k. Test sample on 1% agarose TAE gel (see Section B6 for instructions).

l. Test sample on SDS-PAGE (see Section B5 for instructions). Store at -20 °C.

*Note: Samples can be stored under these conditions for long-term storage (several months).*

5. SDS-PAGE gel electrophoresis (with 1× MES buffer) (Figure 3)

a. Thaw the following on ice:

i. Samples from step B4.

ii. 5× loading dye.

b. Indicate the samples, controls, and ladders used (see Supplementary information, SDS PAGE Electrophoresis [File S5]).

c. Prepare samples in 1.5 mL microcentrifuge tubes accordingly.

*Note: Best practice is to add 5× loading dye, 1× PBS/H_2_O, and then samples (including controls) in order. Do not prepare the ladder or dilute it; the ladder is added by itself when it is time to load the gel.*

d. Vortex briefly and centrifuge on a tabletop centrifuge at 30.73 rcf for 30 s.

e. Heat samples at 95 °C for 5 min on a heat block and cool for 5 min.

f. Prepare Xcell Surelock^TM ^mini-cell with fresh 1× MES buffer and a 12-well NuPAGE^TM^ 10% SDS PAGE gel.

g. Load samples (see Supplementary information, SDS PAGE Electrophoresis [File S5]).

h. Set PowerPac^TM^ to 185 V and 400 mV for 40 min.

i. Remove gel from the box and crack open the mold with a spatula.

*Note: Carefully crack open the mold to avoid tearing the gel.*

j. Transfer gel into a plastic container with a layer of dH_2_O.

k. Briefly rinse and drain gel with dH_2_O.

l. Add safe Coomassie blue stain until gel is covered.

m. Heat in microwave for 1 min.

n. Rock for 5 min at 30 rpm on a compact digital rocker or until cool.

o. Carefully pour the stain back into the Coomassie blue stain bottle.

p. Rinse gel with dH_2_O until the water is not stained blue.

q. Fill the plastic container with dH_2_O and allow the gel to be submerged completely.

r. Add two KimTech wipes on top and microwave for 10 min.

s. Let it cool, replenish with new dH_2_O, and repeat step B5r.

t. Repeat until gel is destained.

u. Transfer gel onto a transparent sheet protector for scanning and record keeping.

6. 1% agarose gel electrophoresis (with 1× TAE buffer)

*Note: All samples, including controls and supernatants, use a volume of 18 µL. Do not dilute.*

a. Weigh 1 g of agarose in a 250 mL Erlenmeyer flask.

b. Add 100 mL of 1× TAE buffer.

c. Heat in a microwave for 1 min and 30 s to dissolve agarose.

d. Let it cool until warm to the touch.

e. Add 1 µL of ethidium bromide and swirl gently.

f. Pour into assembled mold; fill 1/2 way up the comb.

g. Let it cool at 4 °C until the gel sets.

h. Prepare samples (see Supplementary information, Agarose Gel Electrophoresis [File S2]).

i. Add 2 µL of 6× orange loading dye + 18 µL of sample (i.e., controls or supernatants).

*Note: Add the dye and sample together before loading on the gel. A sheet of Parafilm can be used to spot and mix. The dye acts as an anchor to prevent samples from floating out of the gel.*

j. Remove gel from mold, place in a Mini or Wide-Sub^®^ cell GT cell, and fill with 1× TAE buffer to the *max* line.

k. Load samples.

l. Set PowerPac^TM^ to run at 100 V and 400 mV for 20 min.

m. Use a transilluminator for UV light (254/302/365 nm) to visualize the gel.

## Data analysis

The standard operating procedures (SOPs; see supplemental material) were drafted by the Chackerian lab and used to train the corresponding author (Lee) during their postdoctoral training. In 2018, the SOPs were modified to their current state and implemented by the Lee lab to train all new students according to the outlined protocol. Over the last four years, numerous students who primarily identify as high school or undergraduates were trained using the protocol (Table 1). In addition, the protocol was modified in 2020 to train students in chemistry techniques, indicating that the protocol can be utilized in different fields (Table 1). This modified protocol was implemented into the Cultural and Academic Research Experience (CARE) summer program, where it was not emphasized but utilized in the training of large groups of high school students to build foundational skills to prepare for their first research experiences (https://nau.edu/chem-biochem/care/, 2024 [11]). Three cohorts of CARE students have been trained using this protocol.

**Table 1.** Protocol implementation by area of research and academic status (2018–2024)

Category	Biology total	Chemistry total
Masters/post-baccalaureates	2	3
Undergraduates	10	14
High school	3	1
Female	11	12
Underrepresented	8	8
**Current status**		
STEM workforce	4	2
PhD/MD	4	6

## Validation of protocol

This protocol or parts of it have been used and validated in the following research article(s):

• Chackerian. [3]. Virus-like particles: flexible platforms for vaccine development. *Expert Rev Vaccines* 6(3): 381–390. https://doi.org/10.1586/14760584.6.3.381.

Students were trained using this protocol through the Cultural and Academic Research Experience (CARE):

• Chow-Garcia et al. [6]. Cultural identity central to Native American persistence in science. *Cult Stud Sci Educ* 17(2): 557–588. https://doi.org/10.1007/s11422-021-10071-7.

• Lee et al. [12]. Refining a Summer Biomedical Research Training Program for American Indian and Alaska Native (AIAN) Students. *International journal of designs for learning* 9(1): 88. https://doi.org/10.14434/ijdl.v9i1.23049.

## General notes and troubleshooting

**General notes**

1. SOPs are straightforward for synthesizing Qβ VLP. However, some variations, such as the MS2, were observed in the lab. MS2 VLP is sensitive to over-sonication; specifically, excessive or prolonged sonication can damage the structural integrity of the VLP. It is essential to optimize sonication conditions and monitor the process carefully to prevent unintended damage to the VLPs.

2. Plasmids (Qβ and MS2) are not available commercially and must be acquired through the authors as per the protocol.

**Troubleshooting**

**Implementing the multi-phase model**

We identified certain limitations that can occur when using this model.

**Problem 1: Trained student mentors**

For the method to be successful, trained students must be available to act as peer mentors; otherwise, training is reliant on the Principal Investigator. In addition, depending on the student’s level and foundational knowledge, the method was implemented in a ratio of 1:1 for peer mentoring. However, when the peer trainers were more available, such as during the summer months, the method was effective at a 1:3 ratio. It may be difficult to provide adequate training and attention to each student if in higher ratios. The peer mentors must be knowledgeable about the topic to act as a primary source for questions and concerns.

Solution(s): The peer mentors can showcase their knowledge in lab meetings, oral and poster presentations, and one-on-one interaction with the Principal Investigator.

**Problem 2: Timeframe to train new students**

We identified that the timing of when training new students can be a limitation. Often, but not always, we tend to train our new students in the summer months due to more time flexibility. Since most students using this method are undergraduates or high school students, they are more limited in time throughout the school year, leading to a lack of 1:1 consistent mentorship. Finally, maintaining consistency through the training process can lead to more timely and effective results. For example, do not allow for large gaps in time, especially during Phase I, while the students are new.

Solution(s): We recommend at least two academic semesters for the new students or 15 weeks in the summer to showcase their proficiency in Phase II and III. It is recommended that the Principal Investigator check in with the time and productivity of the new students in weekly and/or bi-weekly laboratory meetings.

**Synthesizing Qβ VLP**

**Problem 3: Transformation of C41 *E. coli* cells**

Possible cause(s): Warm or hot inoculating loop kills *E. coli* cells, resulting in no growth on plates.

Solution(s): To ensure proper cooling of the inoculating loop, flame sterilize ~1 min prior to usage. Let it sit near the Bunsen burner as it provides a sterile umbrella to practice aseptic technique.

**Problem 4: VLPs are not extracted during the lysis process**

Possible cause(s): Attributed to insufficient lysing. Under-sonication results in insufficient release of VLPs from cellular matrices.

Solution(s): Allow students to practice with a 50/50 mixture of soap and water to prevent foaming. In addition, to prevent heating, it is advisable to briefly rest samples on ice for 15 s after each sonication cycle. This step underscores the interplay between qualitative art and science. Observe the consistency and discoloration of the sample (expect reduced viscosity and lighting in color).

**Problem 5: VLP samples leaking during the dialysis process**

Possible cause(s): Tearing of dialysis tubes and improper clamping can result in leakage.

Solution(s): Practice preparing dialysis tubes with water and food coloring as your sample. Immerse the dialysis tube in 1× PBS solution to enhance its elasticity and make it less prone to tearing. In addition, fold the ends of the tube before clamping for security.

**Problem 6: Discrepancy in the concentration or dilution of VLP samples**

Possible cause(s): Misapplication of concentration formula (*M1V1* = *M2V2*).

Solution(s): Provide practice problems to ensure proper calculations prior to concentration or dilution of VLPs.

**Problem 7: Samples not collected and/or high impurities on size exclusion chromatography**

Possible cause(s): The gravity column is clogged, blocking proper size exclusion.

Solution(s): Clean (with 1× SCB buffer) or replace Sepharose beads.

**Cleaning steps:**

i. Pipette Sepharose beads into bottle.

ii. Add SCB buffer into bottle and invert gently. Store at 4 °C.

iii. Once beads settle, remove SCB by pipetting. Replenish and repeat the process a few times.

**Synthesizing MS2 VLPs**

**Problem 8: VLPs are not extracted during the lysis process**

Possible cause(s): Attributed to over-lysing. Over-sonication causes heat-induced denaturation and structural degradation of the VLPs.

Solution(s): Briefly rest samples on ice for 15 s after each sonication cycle to prevent heating. This step underscores the interplay between qualitative art and science. Observe the consistency and discoloration of the sample (expect reduced viscosity and lighting in color).

## Supplementary information

The following supporting information can be downloaded here:

1. File S1. Individual Development Plan

2. File S2. Agarose Gel Electrophoresis

3. File S3. Dialysis

4. File S4. Growing, Inducing, and Lysis

5. File S5. SDS-PAGE Electrophoresis

6. File S6. Size Exclusion Chromatography

7. File S7. Transformation
